# Evaluation of the novel avocado/soybean unsaponifiable Arthrocen to alter joint pain and inflammation in a rat model of osteoarthritis

**DOI:** 10.1371/journal.pone.0191906

**Published:** 2018-02-28

**Authors:** Ramin Goudarzi, Allison Reid, Jason J. McDougall

**Affiliations:** 1 Division of Research and Development, Pharmin USA, LLC, San Jose, California, United States of America; 2 Departments of Pharmacology and Anaesthesia, Pain Management & Perioperative Medicine, Dalhousie University, Halifax, Nova Scotia, Canada; University of Calgary, CANADA

## Abstract

**Background:**

Avocado/soybean unsaponifiables such as Arthrocen have been reported to reduce cartilage catabolism and chondrocytic synthesis of inflammatory mediators associated with osteoarthritis (OA). While there is some clinical evidence that avocado/soybean unsaponifiables can reduce OA pain, no preclinical studies have corroborated this observation. The present study determined whether addition of an avocado/soybean unsaponifiable (Arthrocen) to the drinking water of OA rats reduced direct and referred joint pain.

**Methods:**

OA was induced in male Wistar rats by intra-articular injection of sodium monoiodoacetate (MIA: 0.3mg) and animals were allowed to recover for 14 days. Arthrocen was added to the drinking water which was available to animals *ad libitum*. On day 30, joint pain was assessed by dynamic incapacitance while referred pain was determined by von Frey hair algesiometry.

**Results:**

The joint damage induced by MIA injection was severe and was consistent with end-stage OA. Arthrocen consumption (approximately 35 mg/day) attenuated the joint oedema associated with MIA injection. Hindlimb weight bearing also significantly improved in Arthrocen-treated rats (P<0.05); however, von Frey hair mechanosensitivity was unaffected by this treatment.

**Conclusions:**

These data indicate that Arthrocen has the potential to reduce joint inflammation and pain associated with end-stage OA.

## Introduction

Osteoarthritis (OA) is the most prevalent form of arthritis and is heterogeneous in nature. During the development of this disease there is gradual loss of articular cartilage, inappropriate bone remodelling, osteophyte formation, and occasional synovial inflammatory flares. The primary symptoms of OA include joint stiffness, reduced freedom of movement and chronic pain. These debilitating effects lead to reduced mobility and a sharp decline in a patient’s quality of life. In the absence of any effective disease-modifying drugs, the treatment of OA focuses on the much-needed alleviation of joint pain. First line drug therapy uses acetaminophen and non-steroidal anti-inflammatory drugs (NSAIDs); however, the long-term use of these agents can lead to cardiovascular complications, as well as gastrointestinal and renal damage. Opioids are employed as second order analgesics which, although generally effective, can lead to cardiorespiratory problems and have known tolerance issues. Adjunct analgesics for OA patients include the gabapentinoids, serotonin-noradrenaline reuptake inhibitors, and cannabinoids (for review see [1]). These drugs also demonstrate inconsistent efficacy and some negative side-effects which limit their long-term use. Hence, there is a need for safe and effective treatments for the management and alleviation of chronic joint pain.

For non-pharmacological approaches to treating OA, The Osteoarthritis Research Society International (OARSI) recommend land-borne exercise, weight loss, and the use of biomechanical aids (*e*.*g*. splints, braces, orthotics). Natural health products, including dietary supplements, are popular with patients due to their ease of access and perceived margin of safety. Chondroitin sulphate, for example, is a glycosaminoglycan extracted from cartilaginous tissue which in combination with the amino sugar glucosamine has variable effects on joint destruction, inflammation and pain. In a rodent model of arthritis, addition of these agents to a standard diet reduced inflammatory cytokine production, improved cartilage damage, but had no effect on joint pain [2]. In OA patients, chondroitin and glucosamine sulphates have been shown to be both ineffective and effective in reducing joint damage and reported pain [3, 4] which make them unreliable candidates for OA treatment.

In light of the safety concerns associated with some pharmaceutical analgesics, there has been increasing interest in the use of botanical extracts to help treat arthritis. Avocado/soybean unsaponifiables (ASU) are the oily extracts that remain after soap manufacture which include phytosterols, lipophilic vitamins, and triterpinoids. These ASU compounds form the basis of several dietary supplements that have shown some benefit in the treatment of OA [5]. Incubation of bovine articular chondrocytes with ASU (>8.3μg/ml) reduced the expression of inflammatory cytokines and prostaglandin E_2_ [6]. In an ovine model of post-traumatic OA, daily administration of ASU for 6 months resulted in a slight reduction in cartilage proteoglycan loss and subchondral bone sclerosis [7] suggesting that ASU has a slight effect on joint degeneration. The effect of ASU on OA pain is somewhat unclear. In horses, ASU was found to have no effect on experimentally-induced lameness [8]; however, a mild improvement in symptoms has been observed in OA patients taking ASU for several months [9, 10]. The aim of the current study was to evaluate the effect of a commonly used ASU, Arthrocen, on joint pain and inflammation using a rodent model of OA.

## Materials and methods

### Animals

Experiments were carried out on male Wistar rats (200-350g; Charles River Laboratories, Quebec, Canada). Animals were housed two per cage at 20 ± 1°C on a 12h light:dark cycle with free access to food and water. Animals were housed for a minimum of 7 days after arrival before being used in pain behavioural experiments. All animal handling and experimental protocols outlined in this study received prior approval from the Dalhousie University Committee on the Use of Laboratory Animals (Protocol #15–117). Animal were deeply anaesthetised throughout the experiment and ethics followed the guidelines set out by the Canadian Council for Animal Care and ARRIVE.

### Induction of osteoarthritis

Under deep isoflurane anaesthesia (2–4% in 100% O_2_ 1L/min) the right knee joint of the rat was shaved and swabbed with 100% ethanol. Following abolition of the flexor-withdrawal reflex, a 30G needle was inserted into the right knee (stifle) joint through the patellar ligament. With the tip of the needle in the joint cavity, sodium monoiodoacetate (MIA) (3mg/50μl) was injected into the knee and the joint was extended and flexed for 30s to allow distribution of the MIA throughout the synovial space. Animals were allowed to recover for 14 days in order for OA to develop before initiating the pain behavioural tests. Joint diameter and pain behaviour were assessed prior to MIA injection (baseline reading) then on day 14 after OA induction.

### Joint oedema

Joint diameter of rat knees was assessed before MIA injection and at the end of behavioural test days. Under brief isoflurane anaesthesia, a set of digital callipers (VWR, Friendswood, Texas, USA) was oriented anteriorly across the joint line in a medio-lateral plane. The distance between the medial and lateral femoral condyles was measure thrice and an average joint diameter calculated.

### Dynamic incapacitance

The distribution of weight borne by the hind limbs was measured using a dynamic incapacitance system (Bioseb, Florida, USA). Animals were placed in a Plexiglas chamber (24cm long x 24cm wide x 30 cm tall) with unrestricted movement. The floor of the chamber consists of a sensor pad that measures the position, surface area and weight exerted by the hindpaws. A digital video camera mounted at the top of the chamber tracked the animal’s movements throughout a 3min recording period. The sensor pad and camera footage were analysed offline to calculate the weight borne by each limb as well as the surface area of the paws in contact with the sensor pad.

### Von Frey hair algesiometry

The mechanosensitivity of the plantar surface of the ipsilateral hindpaw was assessed using a modification of the Dixon up-down method (Chaplan et al., 1994, J Neurosci Meth 53:55–63). Animals were placed in elevated Plexiglas chambers (30cm long x 9cm wide x 24cm tall) on metal mesh flooring, to allow access to the plantar surface of the paws. After allowing the animal to acclimate until exploratory behaviour ceased (approximately 10min), a von Frey hair, with bending force of 2g, was applied perpendicular to the plantar surface of the hind paw (avoiding the toe pads) until it just bent. The filament was held in place for a total of 3 seconds. If there was a positive response *(i*.*e*. withdrawal, shake or lick of the hindpaw), the next lower strength hair was applied; if there was no response, the next higher strength hair was applied up to a maximum bending force of 15g. After the first difference in response was observed, four more measurements were made and the pattern of responses were converted to a 50% withdrawal threshold calculated using the following formula: 10^[*Xf*+*k*δ]^/10,000; where *Xf* = value (in log units) of the final von Frey hair used, *k* = tabular value for the pattern of the last 6 positive/negative responses, and δ = mean difference (in log units) between stimuli.

### Treatment regimen

Capsules of Arthrocen (300mg, Pharmin USA, CA, USA) were opened and the contents ground into a fine powder with a pestle and mortar. The powder was added to fresh, filtered drinking water and stirred for at least one hour while being slowly heated to 50–60 °C. The concentration of Arthrocen was 30mg/40ml for first 3 days then adjusted to 30mg/45ml for the duration of treatment based on water consumed. Fresh water was prepared every 2 to 4 days. Control animals received fresh, filtered drinking water on the same schedule.

Daily water consumption was monitored for both Arthrocen-treated and control animals and calculated for each water change period as follows: Daily water consumption = total water consumed/(number of days * number of rats in cage). The dose of Arthrocen was calculated as: water consumed (ml/day) * Arthrocen concentration (mg/ml).

Joint diameter and pain behaviour were assessed prior to MIA injection (baseline reading) and then on day 14 after OA induction. Test procedures were then repeated on days 7, 15, 22 and 30 following access to either Arthrocen or regular drinking water.

### Materials and reagents

Sodium monoiodoacetate was supplied by Sigma-Aldrich (St. Louis, MO, USA). Arthrocen capsules were formulated and provided by Pharmin USA (San Jose, CA, USA).

### Statistics

All data passed normality using the Kolmogorov-Smirnov test so were subsequently analysed using parametric statistics. Time courses were analysed using one-way analysis of variance (ANOVA) and treatment effects determined by two-way ANOVA. A Student t-test was used to compare baseline measurement with MIA day 0 to confirm arthritic effects of MIA treatment. All data are presented as means ± S.E.M. for n animals.

## Results

Adding Arthrocen to drinking water did not alter the average daily amount of water consumed (P = 0.1350, two-way ANOVA; *n* = 10–11). Untreated animals drank 53± 1.0 mL/day while Arthrocen treated animals consumed 52±1.0mL/day. Based on the daily water consumption, treated rats received an average dose of Arthrocen of 36±0.5mg/day. Over the 30day test period, Arthrocen-treated rats gained significantly (P = 0.03, two-way ANOVA; *n* = 10–11) less weight than non-treated animals (control: 161±7g; Arthrocen-treated: 147±4g).

### Joint oedema

**Fig 1:**
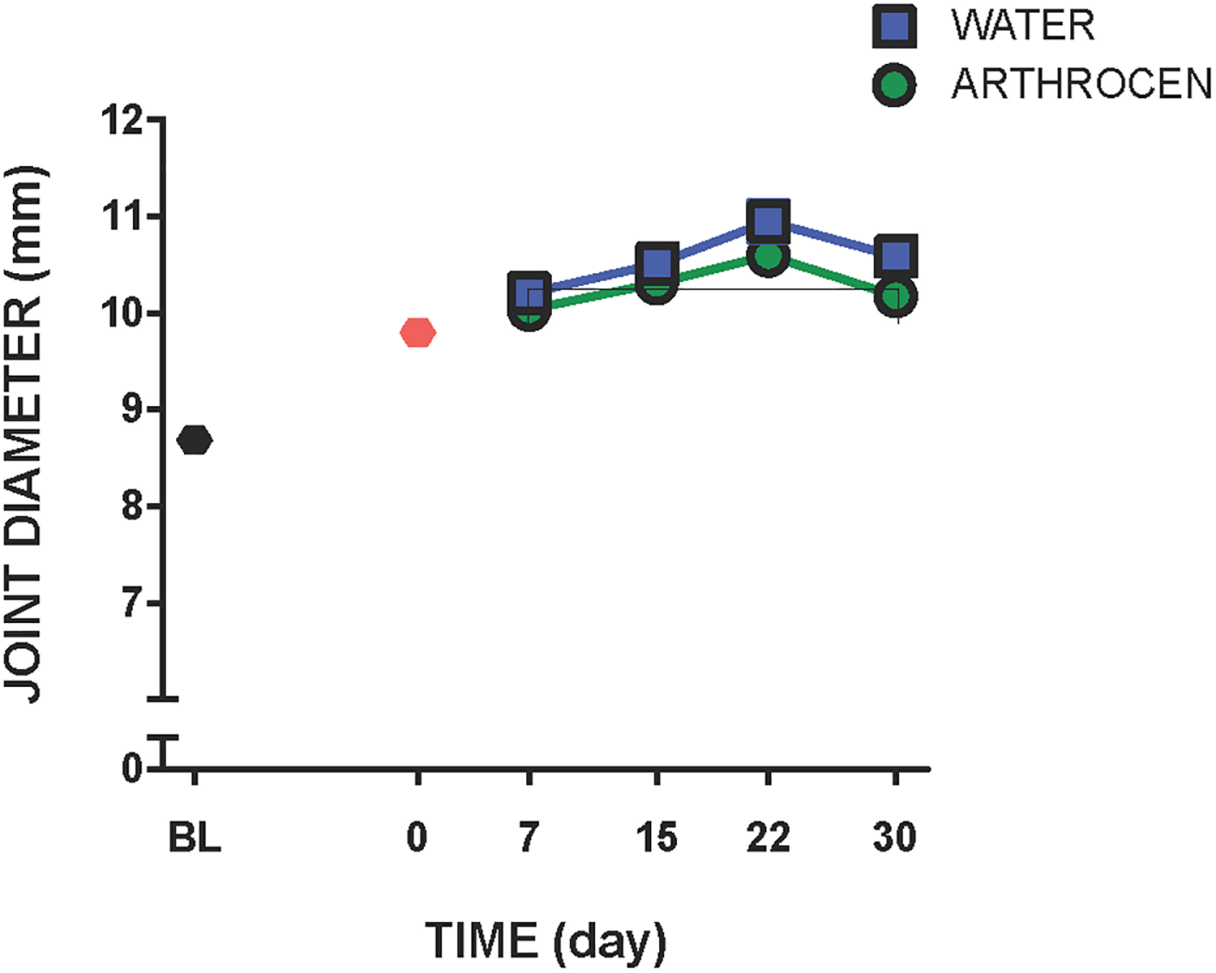
Change in joint diameter in vehicle and Arthrocen-treated OA rats. Over the 30 day test period, Arthrocen attenuated the increase in joint diameter caused by MIA injection. Data are means ±S.E.M.

Fourteen days after intra-articular injection of MIA, knee diameter increased by approximately 13% (Fig 1). Arthrocen significantly attenuated this increase in joint diameter over the 30day treatment period (P<0.01, two-way ANOVA; *n* = 10–11).

### Hindlimb weight bearing

**Fig 2:**
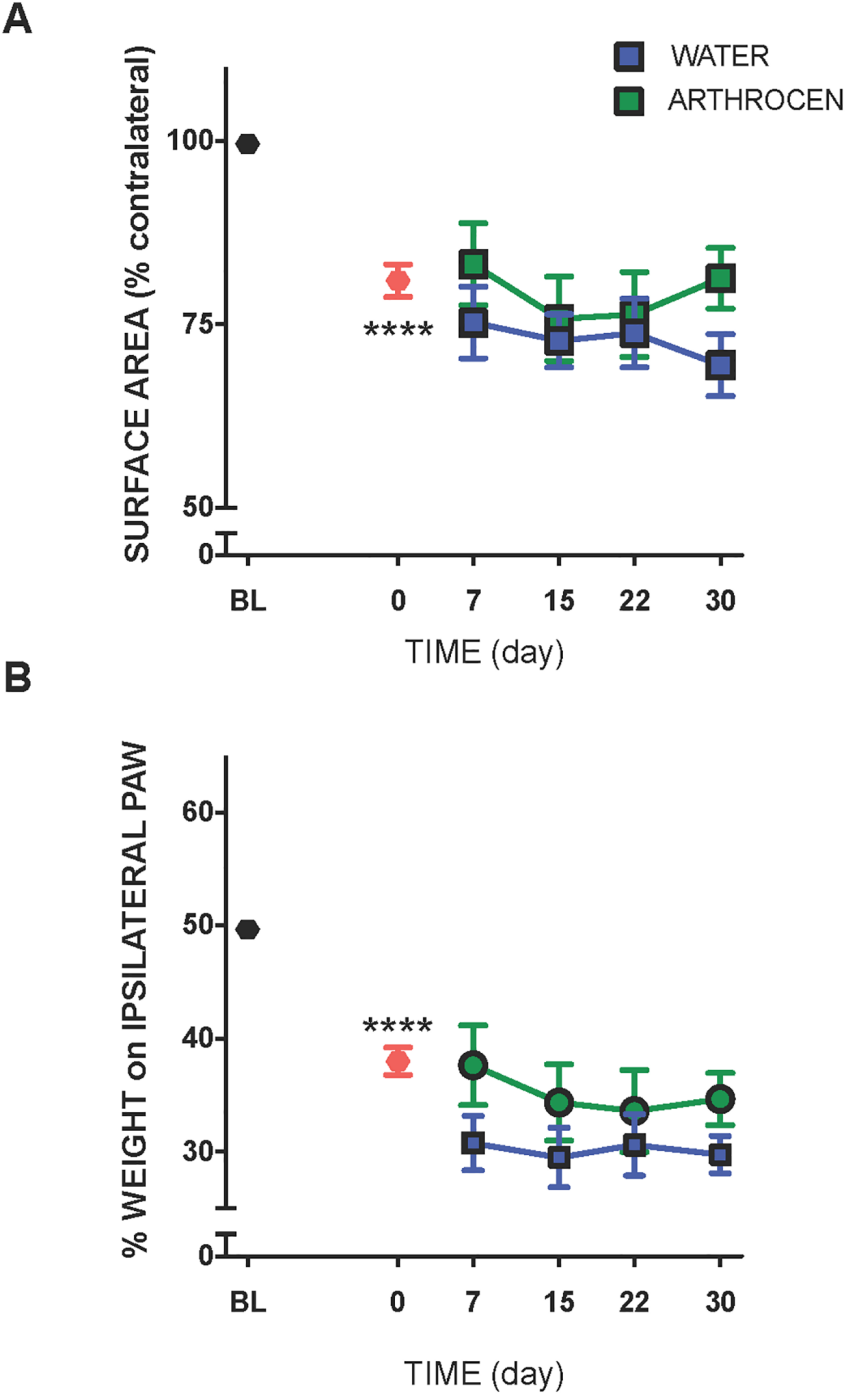
Effect of Arthrocen on hindpaw contact area (A) and hindlimb weight bearing (B) in OA animals. While the extent of hindpaw contact area was unaffected by Arthrocen treatment, the total amount of weight borne by the ipsilateral hindlimb increased over the duration of the treatment period. ****P<0.0001compared to baseline. Data are presented as means ±S.E.M. (*n* = 9–11).

Following MIA treatment, the ground contact area of the ipsilateral hindpaw was significantly reduced (Fig 2A; P<0.0001; *n* = 9–11) indicative of hindlimb pain. This observation was corroborated by the reduction in the amount of body weight borne by the MIA-injected limb (Fig 2B; P<0.0001; *n* = 9–11). Arthrocen did not alter paw surface area (P = 0.07), but did improve ipsilateral weigh bearing (P<0.05).

### Plantar mechanosensitivity

**Fig 3:**
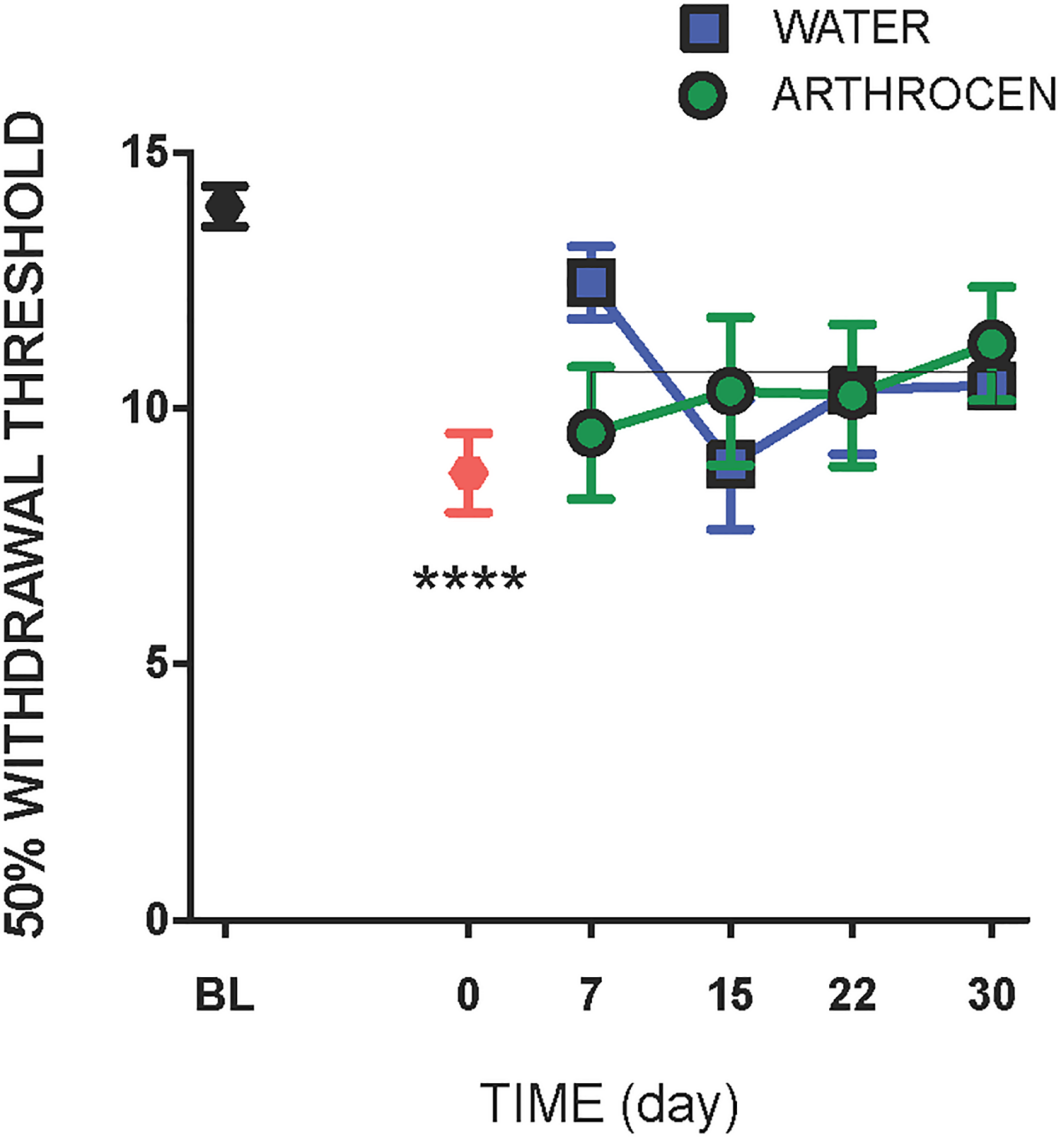
Effect of Arthrocen treatment on secondary allodynia in OA rats. Assessment of referred pain by von Frey hair algesiometry demonstrated that Arthrocen had no effect on hindpaw tactile sensitivity. Data shown as means ±S.E.M.

Compared to baseline, intra-articular injection of MIA caused a significant drop in paw withdrawal threshold (Fig 3; P<0.0001) indicative of secondary allodynia. Arthrocen had no effect on paw mechanosensitivity over the 30 day test period (P = 0.82).

## Discussion

The pharmacological management of OA pain is typically undertreated in most patients. Current drug options carry multiple toxic side-effects which limit their long-term use for chronic OA sufferers. These shortcomings impel patients to seek out natural health products in the belief that they will be safer to use while still being equiefficacious. The present study determined that intra-articular injection of MIA caused a profound pain response as determined by dynamic incapacitance and von Frey hair mechanosensitivity. The magnitude of this pain response is comparable to previous observations using this chemically-induced model of OA [11, 12]. Daily access to an ASU in the drinking water of OA rats caused a small but statistically significant improvement in hindlimb weight bearing. This analgesic effect was apparent at day 7 of Arthrocen treatment and was still apparent at day 30. Interestingly, Arthrocen had no effect on plantar mechanosensitivity throughout this test period. This observation suggests the dietary supplementation with an ASU can relieve pain directly in the OA joint, but does not affect referred pain associated with this disease.

Several clinical studies have demonstrated an analgesic effect of ASUs in OA patients. In a double-blind placebo-controlled trial, Maheu et al. found that treating knee OA patients with an ASU for 6 month led to a decrease in reported pain even 2 months after the conclusion of the study [13]. Similar improvements in degenerative joint pain have been reported in studies involving the hip (10) and temporomandibular joint [14]. In all of these clinical studies, the ASU was administered for several months and at a dose which was 10 times larger than that used in the current study. These differences could account for the more pronounced analgesia described in the clinical trials compared to the relatively mild effect of Arthrocen described here. Future animal studies should consider using a higher dose of Arthrocen over a longer time course.

The mechanism of action for ASU-induced analgesia is unclear. *In vitro* culturing of human and bovine chondrocytes with various ASU formulations have been shown to reduce prostaglandin E2 synthesis and nitric oxide production [6, 15, 16]. These inflammatory mediators are known to sensitize joint afferents and contribute to arthritis pain [1]. Inflammation, however, is only a transient and minor component of the MIA model of OA and typically resolves by day 14. This could explain why Arthrocen was only moderately effective at reducing joint pain in this particular model. Future studies are required to test Arthrocen in preclinical models of OA in which there is a more pronounced inflammatory component.

## Conclusions

In summary, daily administration of Arthrocen caused a modest improvement in hindlimb weight bearing and associated pain, but not secondary allodynia. Further studies using a higher dose of Arthrocen for a longer time course are required to confirm the maximum level of analgesia of this ASU for the treatment of OA joint pain.
